# A Rare Case of Oral-Maxillary NUT (Nuclear Protein in Testis) Carcinoma With Diffuse Osseous Metastasis and Hypercalcemia of Malignancy

**DOI:** 10.7759/cureus.82244

**Published:** 2025-04-14

**Authors:** Layla Abdul Jabbar, Lucia Soca, Cecilia Clement, Suimin Qiu, Carlos Dostal

**Affiliations:** 1 Internal Medicine, University of Texas Medical Branch at Galveston, Galveston, USA; 2 Pathology, University of Texas Medical Branch at Galveston, Galveston, USA

**Keywords:** endocrine complications, hypercalcemia of malignancy, nut carcinoma, osteolytic metastasis, paraneoplastic syndrome

## Abstract

Nuclear protein in testis (NUT) carcinoma is a rare and aggressive cancer arising from genetic rearrangements of the *NUTm1* gene. This leads to tumorigenesis, which commonly affects midline structures, including the head, neck, and thorax. It may originate from non-cutaneous epithelial tissues and resemble poorly differentiated squamous cell carcinomas. NUT carcinoma (NC) can typically be consistently identified through immunostaining for the NUT protein. Unfortunately, most cases are detected at an advanced stage, leading to unfavorable outcomes. The rarity of NC, combined with its aggressive behavior, late presentation, and limited understanding of its pathogenesis, presents considerable diagnostic and therapeutic challenges. We present and discuss our case of a 48-year-old male with NC of oral-maxillary origin with diffuse osteolytic metastasis. Uniquely, this is the first report of adult NC with severe hypercalcemia of malignancy (20.1 mg/dL) resistant to an aggressive multimodal treatment approach. We underscore the urgent need for the timely diagnosis of NC and the development of effective interventions for improving the outcome of this devastating malignancy.

## Introduction

Nuclear protein in testis (NUT) carcinoma is an aggressive subtype of squamous cell carcinoma arising from a chromosomal translocation involving the *NUTm1* gene. This genetic alteration, which is driven by a chromosomal translocation involving the *NUTm1* gene, most commonly results in a BRD4-NUT fusion oncogene. This fusion protein blocks differentiation and promotes tumor growth by inducing widespread chromatin acetylation and oncogenic gene expression. The alteration appears sporadic, with no known environmental or inherited risk factors identified to date, leading to aberrant squamous cell growth that can occur in any part of the body, most often described in the midline structures, such as the head, neck, and thorax [1]. The clinical presentation of NUT carcinoma (NC) varies depending on the affected body regions. General signs and symptoms include weight loss, fatigue, cough, generalized pain, and local mass effect depending on the site involved.

NC affects patients of both sexes equally, with a median reported age of 24 with most reports in teens and young adults [2,3]. Diagnosis of NC is limited by institutional resources and a lack of awareness surrounding the condition. Increased awareness of NC and access to molecular diagnostics likely contribute to an increased incidence of NC diagnosis. However, the exact incidence and prevalence of NC remain unknown [4].

NC exhibits highly nonspecific morphological and clinical features overlapping with other carcinomas; as a result, it is not categorized through traditional methods such as site of origin or tissue of origin; instead, NC is diagnosed via NUT immunohistochemical staining using an antibody detecting NUT protein expression with 100% specificity and 87% sensitivity. Additional molecular analysis is recommended in the initial diagnostic evaluation to identify the specific fusion gene product involved in tumor formation. This is clinically significant beyond diagnostic purposes as different fusion partners to NUT are associated with different prognostic outcomes [5-7]. For instance, non-thoracic primary tumors with BRD3-NUT or NSD3-NUT have the longest survival, followed by the non-thoracic primary tumors with BRD4-NUT. Even so, 70% of NC cases involve BRD4-NUT while the remaining 30% of cases involve BRD3-NUT and NSD3-NUT, among other possible gene fusion variants [8]. This might explain one aspect of the aggressiveness of the tumor at diagnosis, since the most identified genetic variant (i.e., BRD4-NUT) has the worst prognostic outcomes. Furthermore, location and tumor spread at the time of diagnosis also affect prognosis and survival time. Independent of tumor genetics, thoracic primary tumors and the presence of metastasis are associated with the worst overall survival. Ultimately, primary tumor location, stage at diagnosis, and genetic rearrangement influence survival times and shape management strategies at the time of diagnosis [9].

Overall, the NC prognosis is poor, with a mean survival time of approximately seven months from the time of diagnosis. Treatment is challenging as there are no specific guideline-directed therapies to target NC. Management typically involves a multimodal approach with surgical resection and radiation therapy as the cornerstones of treatments [10]. Chemotherapy with standard cytotoxic regimens is often used with no clearly effective treatment regimen identified at the time of this report. Ongoing phase I/II clinical trials are exploring targeted therapies to improve treatment options, combining bromodomain extra-terminal (BET) inhibitors and histone deacetylase inhibitors to displace BRD4-NUT fusion proteins from chromatin and thereby help to arrest cancer growth. Most recently, a high PD-L1 expression was identified in NC, suggesting a role for immune checkpoint inhibitors. In fact, positive responses regardless of PD-L1 positivity have been reported; however, more data are required to substantiate the findings [10].

In this case report, we present a unique case of a middle-aged male with a right facial mass found to have NC complicated by osseous metastasis and severe hypercalcemia of malignancy (HCM).

## Case presentation

A 47-year-old incarcerated African American man with a past medical history of hypertension presented to the Oral-Maxillofacial Surgery (OMFS) clinic with four months of a worsening painful right maxillary mass. At the time of presentation, the patient further endorsed these associated symptoms, including facial swelling, numbness, dysphagia, night sweats, palpitations, and weight loss. Past surgical, family, and social history were unremarkable. His oral examination revealed a right exophytic maxillary mass. An orthopantomogram was concerning for destruction of the right maxillary sinus features. OMFS considered admission for biopsy; however, pre-op labs included severe hypercalcemia (20.1 mg/dL), and the patient was admitted to internal medicine for further evaluation.

**Figure 1:**
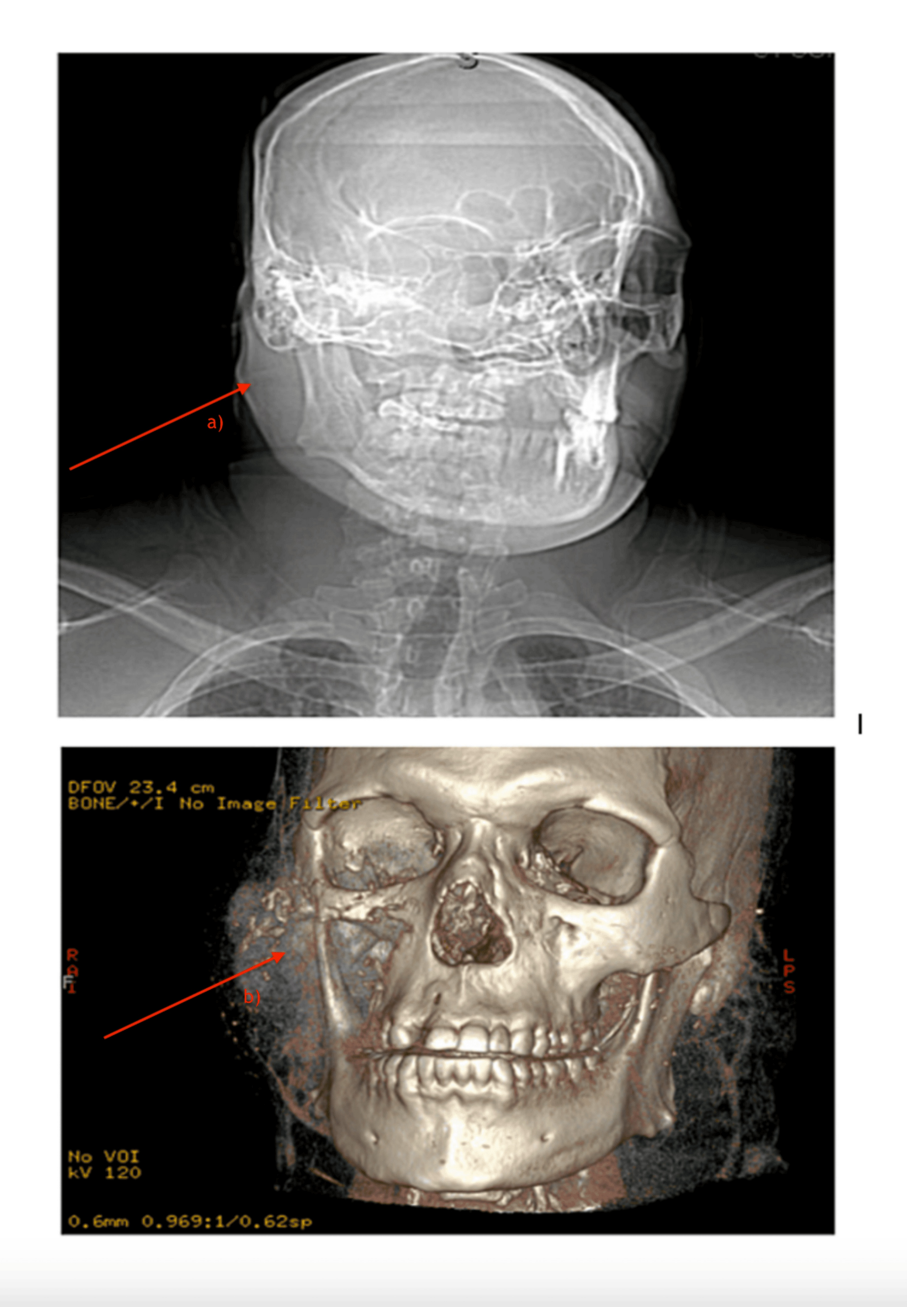
Maxillofacial and mandibular computed tomography with contrast (a) Illustration of an 8.5 x 7.7 x 9 cm soft-tissue mass (later confirmed as NUT [nuclear protein in testis] carcinoma) with (b) extensive tumor invasion resulting in partial destruction of the right masticator, right maxillary sinus, right inferior orbit, and right pterygoid regions.

**Figure 2:**
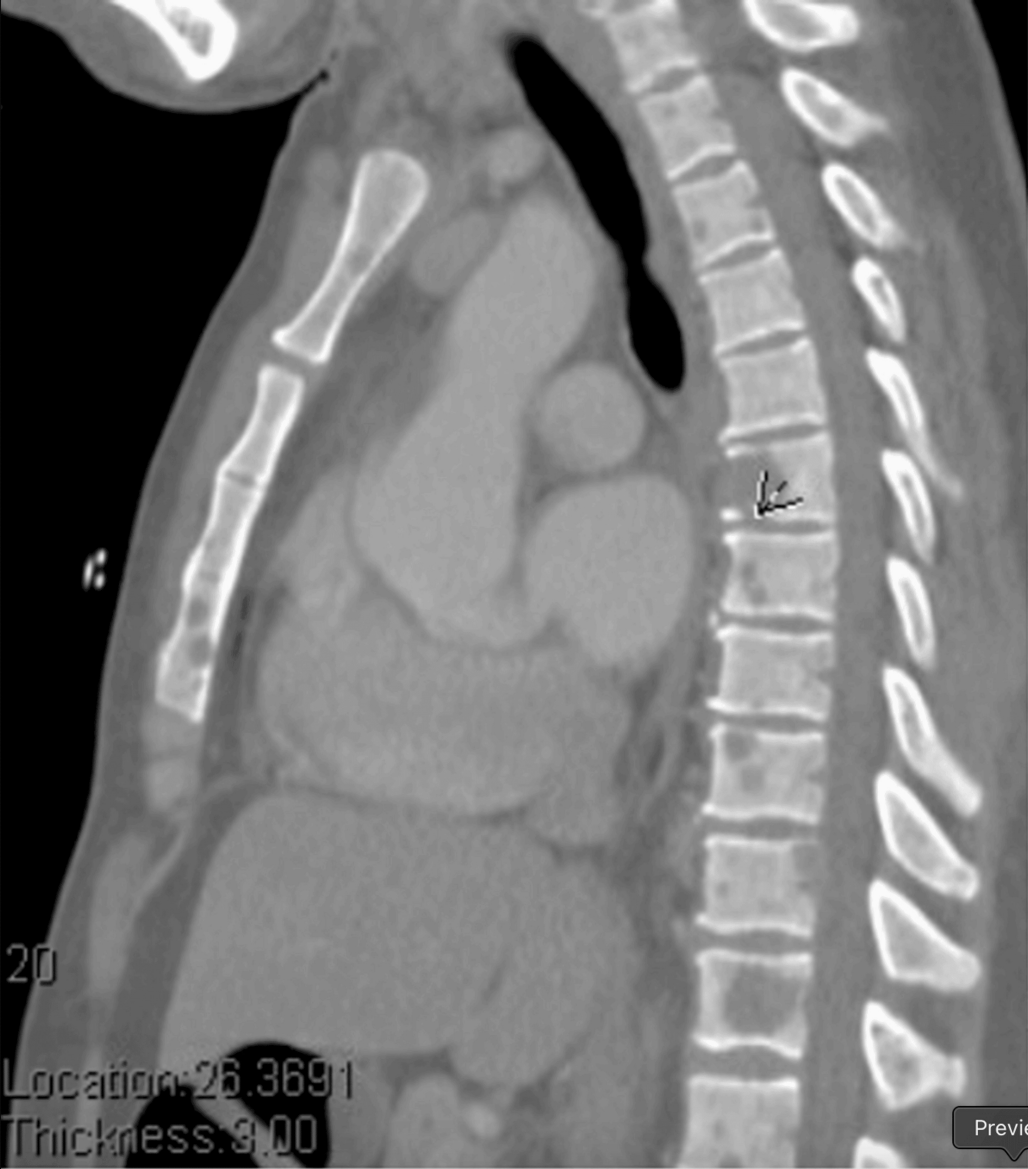
Computed tomography with contrast of the patient's chest The image reveals diffuse lytic lesions throughout the visible bones of the chest wall. One of the dominant lytic lesions at the T6 vertebral body (arrow) is associated with a small non-displaced fracture. Another large lytic lesion replaces much of the T11 vertebral body including the right posterior elements.

**Figure 3:**
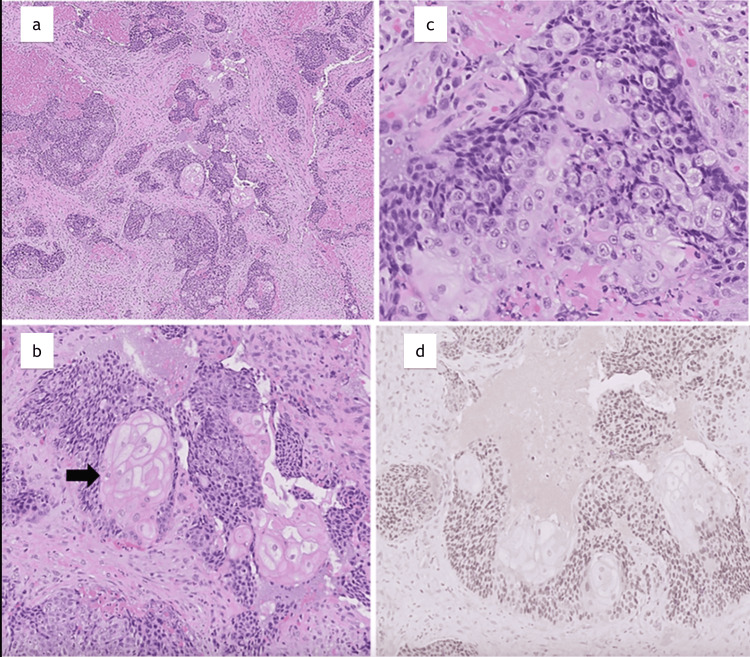
Histological features of the patient's NUT (nuclear protein in testis) carcinoma presentation (a) Clusters of highly infiltrative malignant cells (H&E, 50×). (b) Primitive small- to medium-sized cells with dark nuclei and scant cytoplasm, admixed with “fried egg” appearance with abundant pink cytoplasm and variable prominent nucleoli (H&E, 400×). (c) Foci of abrupt squamous differentiation (black arrow) with clear to eosinophilic cytoplasm (H&E, 200×). (d) Immunohistochemistry with strong nuclear staining in more than 90% of tumor cells (NUT immunostain, NUT (1:25) on Leica Bond iii platform, C52B1 -clone, cell signaling, catalogue number 3625S, 200×).

On admission, vital signs were unremarkable. The patient was alert, ambulatory, and with obvious right-sided facial swelling, V2 paresthesia, right eye proptosis, right submandibular lymphadenopathy, and hyperreflexia of the upper extremities. Contrast computed tomography (CT) imaging revealed an 8.5 x 7.7 x 9 cm mass (Figure 1a) involving the right masticator, right maxillary sinus, right inferior orbit, and right pterygoid regions with orbital invasion (Figure 1b). Widespread osseous lytic lesions were found on staging CT chest (Figure 2) and abdominal imaging. Together, this was concerning for diffuse metastatic disease with a suspected oral-maxillary primary tumor. Biopsy of the right maxillary mass was remarkable for poorly differentiated squamous cell carcinoma versus NC (Figure 3a-3c). Positive immunohistochemistry (IHC) with NUT protein expression in >90% of nuclei confirmed the diagnosis of NC (Figure 3d).

**Figure 4:**
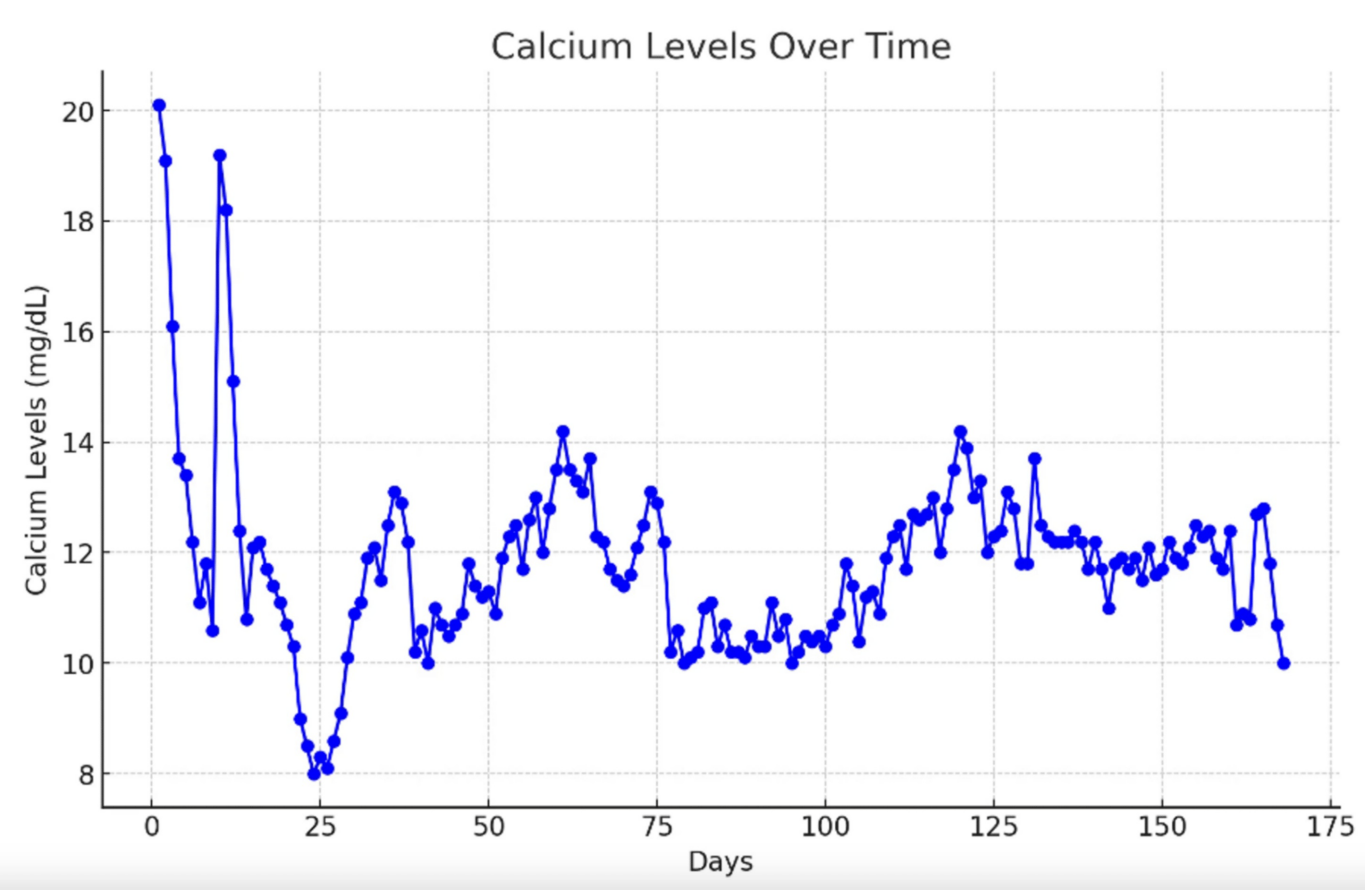
Calcium graph of the progression of the patient's levels The graph illustrates the progression of serum calcium levels in our patient with NUT (nuclear protein in testis) carcinoma, a rare and aggressive malignancy. The patient exhibited severe hypercalcemia of malignancy, a known paraneoplastic phenomenon. Despite initial stabilization attempts with intravenous fluids, bisphosphonates, and calcitonin, calcium levels remained refractory, reflecting the aggressive nature of the underlying malignancy.

The patient was not a surgical candidate due to diffuse metastatic disease and was instead initiated on palliative chemotherapy with 750 mg [area under the curve (AUC) = 5] carboplatin and 175 mg/m^2^ paclitaxel. Laboratory studies were consistent with HCM [parathyroid hormone (PTH) = 6.5 pg/mL, PTH-related peptide (PTHrp) = 18.8 pmol/L, vitamin D = 15 ng/mL], and calcium was corrected with intravenous fluids, calcitonin, and zoledronic acid. He was discharged and received palliative radiation directed to the maxillary mass at 300 cGy for 10 fractions. Surveillance imaging, about two months after radiation and two cycles of chemotherapy, was favorable for about a 66% interval decrease in the maxillary tumor size. Even so, body imaging demonstrated interval worsening of osseous metastasis. Treatment failure was further supported by PTHrp increasing from 18.8 pmol/L to 45.7 pmol/L. Three months after diagnosis, the patient was bed-bound with right-arm paralysis and pathological compression fractures of C7, T6, and T11. He completed 10 additional fractions of palliative radiation at 300 cGy each to the C7, T6, and T11 lesions. He was no longer a chemotherapy candidate due to poor functional status. He suffered numerous re-admissions for complications, including recurrent HCM (Figure 4), severe neutropenia, recurrent sepsis with respiratory failure, pneumonia, and recurrent *Klebsiella* bacteremia.

## Discussion

There are no specific guideline-directed therapies for NC. Management typically involves a multimodal approach with surgical resection, chemotherapy, and/or radiation therapy. Due to its rarity and highly variable treatment approaches, NC remains an extremely challenging cancer to manage [11]. Our 47-year-old patient’s clinical course suggests that his tumor was sensitive to radiation therapy but resistant to systemic chemotherapy, as his primary facial tumor responded dramatically while diffuse osseous metastasis continued progressing. Indeed, the only documented survivor of NC was treated with aggressive surgical management and adjuvant chemoradiation therapy [11].

HCM is the most common paraneoplastic syndrome, occurring in 20% to 30% of cancer patients. It is an oncologic emergency owing to severe complications, including coma and renal failure. HCM is associated with poor prognosis despite active treatment and a mean survival of 1-3 months. Our case developed refractory HCM despite radio-chemotherapy and aggressive therapy, including weekly doses of zoledronic acid, ultimately necessitating treatment with denosumab [12]. HCM in NC was first reported in an 11-year-old female. The mechanism of HCM herein is related to tumor-derived PTHrP secretion; however, concurrent infiltrative osteolysis is possible with severe bone metastases. To our knowledge, this is the first report of severe recurrent HCM in adult NC [13].

## Conclusions

NC remains a perplexing and formidable challenge in the realm of oncology. Its challenging clinical course and rapid progression call for an innovative approach to treatment. As highlighted in this case study, combining chemotherapy, radiation therapy, and palliative measures offers little hope for extended survival. Thus, early detection and heightened awareness are essential, with aggressive surgical management offering the best prognosis. Ongoing research and clinical trials hold promise for novel therapeutics to improve outcomes for patients confronted with this life-threatening malignancy. The path to treating NC is challenging, but through shared knowledge and continuous research, we can aspire to provide better solutions and brighter prospects for those affected by this rare disease.
